# Selective expansion of gut antibiotic resistome and underlying pathways involved in type 1 diabetes

**DOI:** 10.1002/imo2.70007

**Published:** 2025-03-06

**Authors:** Guozhu Ye, Yifang Duan, Haining Huang, Guoyou Chen, Minghui Li, Ricardo David Avellán‐Llaguno, Qiansheng Huang

**Affiliations:** ^1^ Xiamen Key Laboratory of Indoor Air and Health, Center for Excellence in Regional Atmospheric Environment, Key Lab of Urban Environment and Health, Institute of Urban Environment Chinese Academy of Sciences Xiamen China; ^2^ College of Pharmacy, Daqing Campus Harbin Medical University Daqing China

## Abstract

Selective expansion of gut antibiotic resistance genes (including glycopeptides, tetracyclines, macrolide‐lincosamide‐streptogramins, and beta‐lactams) was observed in type 1 diabetic rats. Interestingly, a similar expansion of gut antibiotic resistome occurred in diabetic patients. Selective expansion of gut antibiotic resistome was correlated with hyperglycemia and gut microbial community (particularly order *Lactobacillales*, such as species *Lactobacillus johnsonii*, *Limosilactobacillus urinaemulieris*, and *Streptococcus hyointestinalis*). Furthermore, activation of peptidoglycan biosynthesis and beta‐lactam resistance, disturbances in vancomycin resistance, and activation of antimicrobial peptide resistance, lantibiotic biosynthesis and immunity, and transport of antibiotics, peptides, and amino acids were involved in selective expansion of gut antibiotic resistome in type 1 diabetic rats.

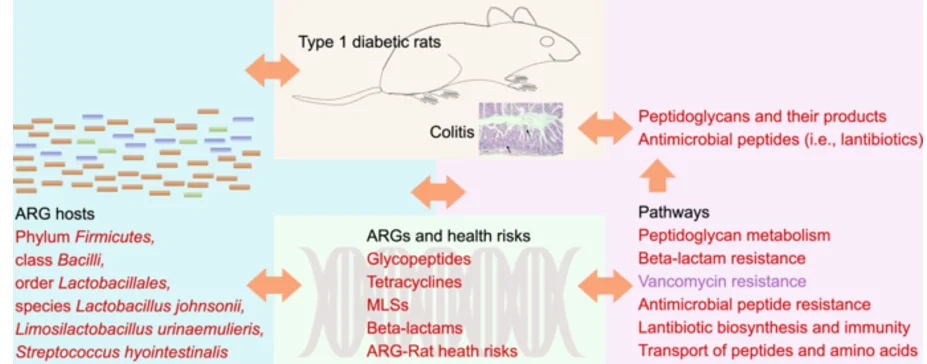

To the editor,

As emerging pollutants, antibiotic resistance genes (ARGs) have been recognized as global public health issues, which pose increasing health hazards to human and the environment [1]. The global antibiotic crisis in treating deadly microbial infections can be largely attributed to the selective collection of ARGs, known as the resistome, in gut microbiota [2]. Type 1 diabetes (T1D), a major type of diabetes, is characterized by autoimmune β‐cell destruction, and resultant insulin deficiency and hyperglycemia [3]. Notably, T1D often occurs in childhood and is growing rapidly [4]. Accordingly, research on T1D is extremely urgent and significant. However, interactions between gut microbial antibiotic resistome and T1D are unclear.

Gut microbiota mediates the occurrence and progression of diseases, and acts as key reservoirs of ARGs and hotspots for ARG acquisition and dissemination [2, 5, 6, 7]. Besides, ARGs mediate antibiotic metabolism, induce antibiotic target alterations, and regulate microbial quorum‐sensing strategies and even host responses, thus affecting gut microbial ecological dynamics [6, 8]. Notably, selective collection of ARGs in gut microbiota and microbial adaptation strategies to antibiotics occur before antibiotic exposure [6, 9]. Additionally, inflammation and oxidative stress could promote the selection of specific microbes and ARGs by mediating horizontal and/or vertical gene transfer and microbial and/or host metabolism [5, 10]. Currently, whether selective collection of gut antibiotic resistome occurs with T1D occurrence and progression is unknown. Meanwhile, the effects of T1D on ARG emergence and dissemination, roles of gut antibiotic resistome in T1D occurrence and progression, and underlying ARG hosts and pathways are still undefined. Thus, we hypothesized that selective collection of gut antibiotic resistome would occur upon T1D. Subsequently, metagenomics was employed to unveil distinctive antibiotic resistome and its health risks, underlying ARG hosts and pathways. Furthermore, the selective expansion of gut antibiotic resistome and its health effects were further examined in diabetic patients.

## 1 RESULTS AND DISCUSSION

### Selective expansion of gut antibiotic resistome and its health risks in T1D rats

The volume of islets and the number of endocrine cells were decreased in T1D rats, and serum glucose reached 21.82 mmol/L in T1D rats, which was 2.26 times that in the normal group (Figure S1A, S1B and Table S1). These data demonstrated the successful establishment of the diabetic model. Besides, a large amount of secretions and inflammatory cell infiltration in the glandular duct, and inflammatory cells between the glandular tubes were observed, which indicated colitis in T1D rats (Figure S1C).

**FIGURE 1:**
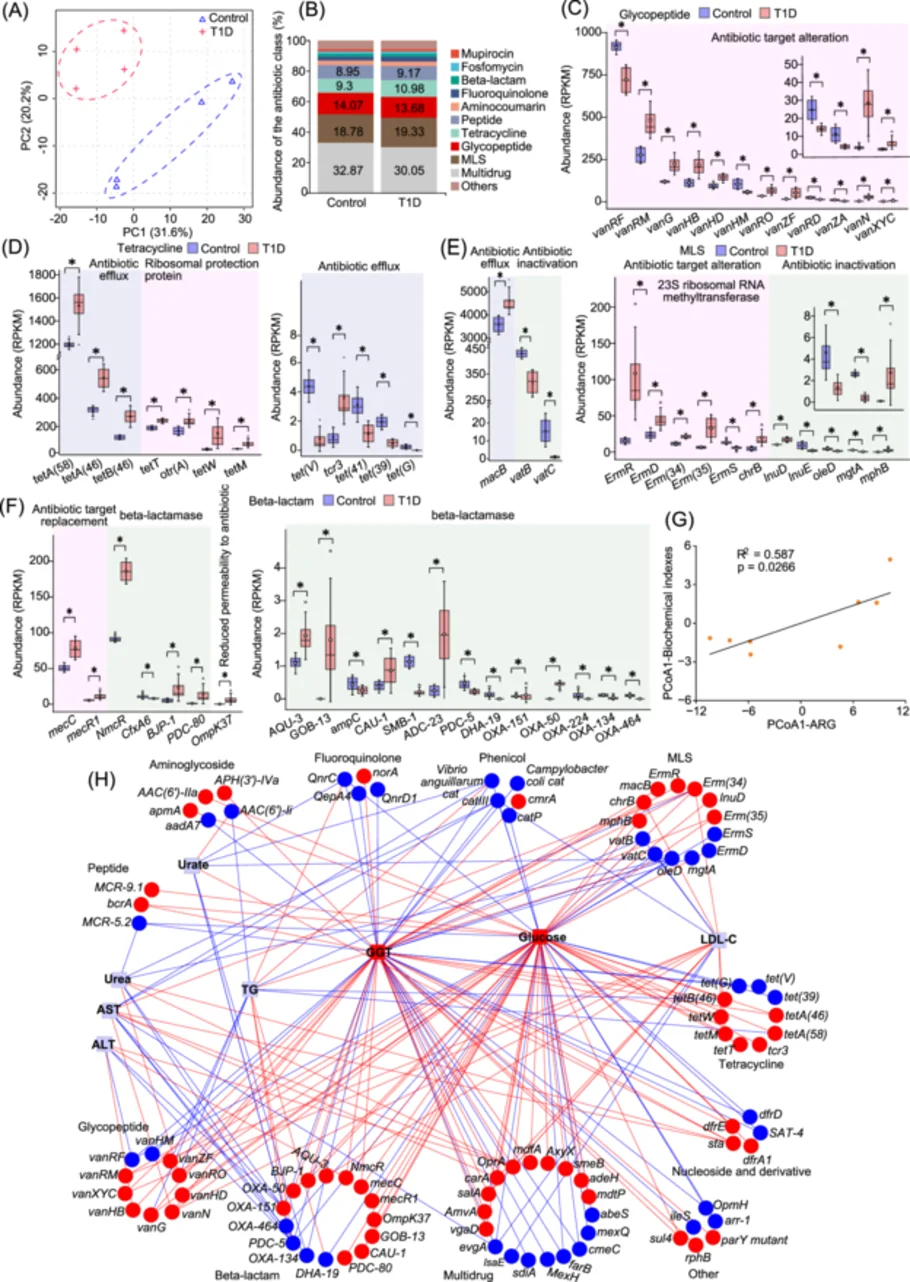
Selective expansion of gut antibiotic resistome and its health risks in type 1 diabetes (T1D) rats. (A) Changes in profiles of gut antibiotic resistome. (B) Changes in compositions of gut microbial antibiotic resistance gene (ARG) types. Accumulation of glycopeptide (C), tetracycline (D), MLS (E), and beta‐lactam (F) ARGs in gut microbiota in T1D rats. *, *p* < 0.05, two‐tailed Man–Whitney *U* test. RPKM, gene abundances defined as reads per kilobase million. (G) Regression analysis of gut antibiotic resistome and serum biomedical indexes. The first principal component of principal coordinate analysis (PCoA) was used. (H) Correlation network analysis of gut microbial ARGs and serum biomedical indexes. The Spearman correlation coefficient was lower than −0.7 or greater than 0.7. Squares/circles, biochemical indexes/ARGs; blue/red lines, negative/positive correlations; blue/red shapes, significantly decreased/increased in T1D rats; gray shapes, not significantly altered in T1D rats. AST, aspartate transaminase; ALT, alanine transaminase; GGT, gamma‐glutamyl transferase; LDL‐C, low‐density lipoprotein cholesterol; TG, triglyceride.

The profiling of gut antibiotic resistome in T1D rats differed greatly from that in normal rats (Figure 1A). Multidrugs, macrolide‐lincosamide‐streptogramins (MLSs), glycopeptides, tetracyclines, and peptides were predominant ARG types, accounting for >83.0% of the total abundance in rats (Figure 1B). Tetracyclines, peptides, antibiotic target alteration, and antibiotic target protection were increased in T1D rats (Figure S2A,B). Totally, 125 ARGs were significantly altered in T1D rats, including 30 multidrugs, 20 beta‐lactams, 14 MLSs, 12 glycopeptides, 12 tetracyclines, 9 aminoglycosides, and others (Figures S2C, S3 and Table S2).

Notably, 8 of the 12 significantly altered glycopeptide ARGs were increased, indicating accumulation of glycopeptide ARGs in T1D rats (Figure 1C). With regard to tetracycline ARGs, high‐abundance ARGs involved in efflux pumps or ribosomal protection proteins, such as *tetA(58)*, *tetA(46)*, *tetM*, and *tetT* were significantly increased, while low‐abundance efflux pump genes were mainly decreased in T1D rats (Figure 1D). Considering the above changes and abundances of ARGs, it could be concluded that tetracycline ARGs accumulated in T1D rats. MLS ARG accumulation also occurred in T1D rats (Figure 1E). High‐abundance ATP‐binding cassette transporter gene *macB* and 23S ribosomal RNA methyltransferase genes, such as *ErmR*, *ErmD*, and *Erm(35)*, were increased, while most low‐abundance ARGs involved in antibiotic inactivation (such as *lnuE*, *oleD*, and *vatC*) were decreased in T1D rats (Figure 1E). Regarding beta‐lactam ARGs, high‐abundance beta‐lactam ARGs involved in encoding methicillin‐resistant penicillin‐binding protein 2 or beta‐lactamases (such as *mecC*, *mecR*, and *NmcR*) and *OmpK37* encoding porins were increased in T1D rats (Figure 1F). These data indicated that beta‐lactam ARGs accumulated in T1D rats.

We discovered high correlations among gut microbial ARGs, which suggested that changes in gut antibiotic resistome in T1D rats were systemic and might be mediated by some key signaling and/or pathways (Figure S4). Besides, strong correlations between gut antibiotic resistome and serum biochemical indexes were found, especially those of hyperglycemia with glycopeptides, tetracyclines, MLSs, beta‐lactams, and multidrugs in T1D rats (Figure 1G,H). These data suggested potential health effects of gut antibiotic resistome expansion on hosts. ARGs, especially those involved in two‐component systems (e.g., CpxR signaling), can sense changes in intracellular and extracellular factors and respond by modulating metabolism (e.g., cell envelop metabolism). In turn, antibiotic ressitome is influenced by the microbial community, and metabolism of microbes and hosts. Therefore, underlying ARG hosts and pathways were explored.

## 2 SELECTIVE EXPANSION OF GUT ANTIBIOTIC RESISTOME AND ITS HEALTH RISKS IN DIABETIC PATIENTS

Similarly, the selective expansion of gut antibiotic resistome occurred in patients with gestational diabetes mellitus (GDM) or type 2 diabetics (T2D, Figure S5) [11, 12]. Tetracyclines, MLSs, beta‐lactams, aminoglycosides, and bacitracins were predominant ARG types in GDM, accounting for more than 96% of the total. Of the 28 ARGs (belonging to aminoglycosides, beta‐lactams, MLSs, multidrugs, or tetracyclines) significantly accumulating in GDM, 25 were significantly correlated with higher GDM risks. Additionally, multidrugs, fluoroquinolones, fusidic acids, elfamycin, and beta‐lactams were predominant ARG types, accounting for more than 70% of the total in T2D patients (Figure S5). Among the 62 ARGs with significant alterations in T2D patients, 48 were increased, demonstrating gut antibiotic resistome expansion in T2D patients, such as accumulation of beta‐lactams, fluoroquinolone‐resistant gyrA/B, multidrugs, fosfomycin, and isoniazids (Figure S5 and Table S3). In addition to beta‐lactams, major facilitator superfamily antibiotic efflux pump genes belonging to multidrugs accumulated in T1D rats and T2D patients [12]. Notably, strong correlations of gut antibiotic resistome with clinical factors (especially fasting blood glucose, glycosylated hemoglobin HbAlc, triglycerides, and age) were observed in T2D patients. Besides, among 12 ARGs significantly associated with higher T2D risks, 7 of which were fluoroquinolone‐resistant *gyrA* or *parC*.

## 3 SPECIES CONTRIBUTIONS OF GUT MICROBIOTA TO ANTIBIOTIC RESISTOME IN T1D RATS

We found that changes in gut microbial community (from the phylum to species) were significantly correlated with those in antibiotic resistome in T1D rats, and that the phylum *Firmicutes* contributed most to antibiotic types, followed by *Bacteroidota* and *Actinobacteria* (Figures S6 and S7A). Additionally, multidrugs, MLSs, glycopeptides, tetracyclines, and peptides were the main antibiotic types of phyla *Firmicutes*, *Bacteroidota*, *Proteobacteria*, and *Actinobacteria* (Figure S7B). Moreover, contributions of *Firmicutes* to tetracyclines, peptides, mupirocins, diaminopyrimidines, and triclosans were significantly increased in T1D rats (Figure S7C).

In total, 125 species' contributions at the species level to antibiotic types were significantly altered (Figure S8). Notably, 32 of 52 species contributions from the phylum *Bacteroidota* were significantly decreased, while 46 of 65 species contributions from the phylum *Firmicutes* were increased, suggesting that selective expansion of gut antibiotic resistome might be mainly attributed to the contribution of *Firmicutes*. As shown in Figure S9, species *Lactobacillus johnsonii*, *Limosilactobacillus urinaemulieris*, and *Streptococcus hyointestinalis*, belonging to order *Lactobacillales* (class *Bacilli*, phylum *Firmicutes*), were key contributors to glycopeptides, tetracyclines, multidrugs, and MLSs in T1D rats. Besides, species *Lactobacillus johnsonii* and *Muribaculaceae bacterium* Isolate‐013 (NCI) were key contributors to beta‐lactams in T1D rats (Figure S9). Moreover, species *Lactobacillus johnsonii*, *Limosilactobacillus urinaemulieris*, and *Muribaculaceae bacterium* were key contributors to aminoglycosides in T1D rats (Figure S9). Taken together, *Lactobacillus johnsonii* was the species contributing most to glycopeptides, tetracyclines, multidrugs, MLSs, beta‐lactams, and aminoglycosides in T1D rats.

## 4 MOLECULAR PATHWAYS INVOLVED IN SELECTIVE EXPANSION OF GUT ANTIBIOTIC RESISTOME IN T1D RATS

**FIGURE 2:**
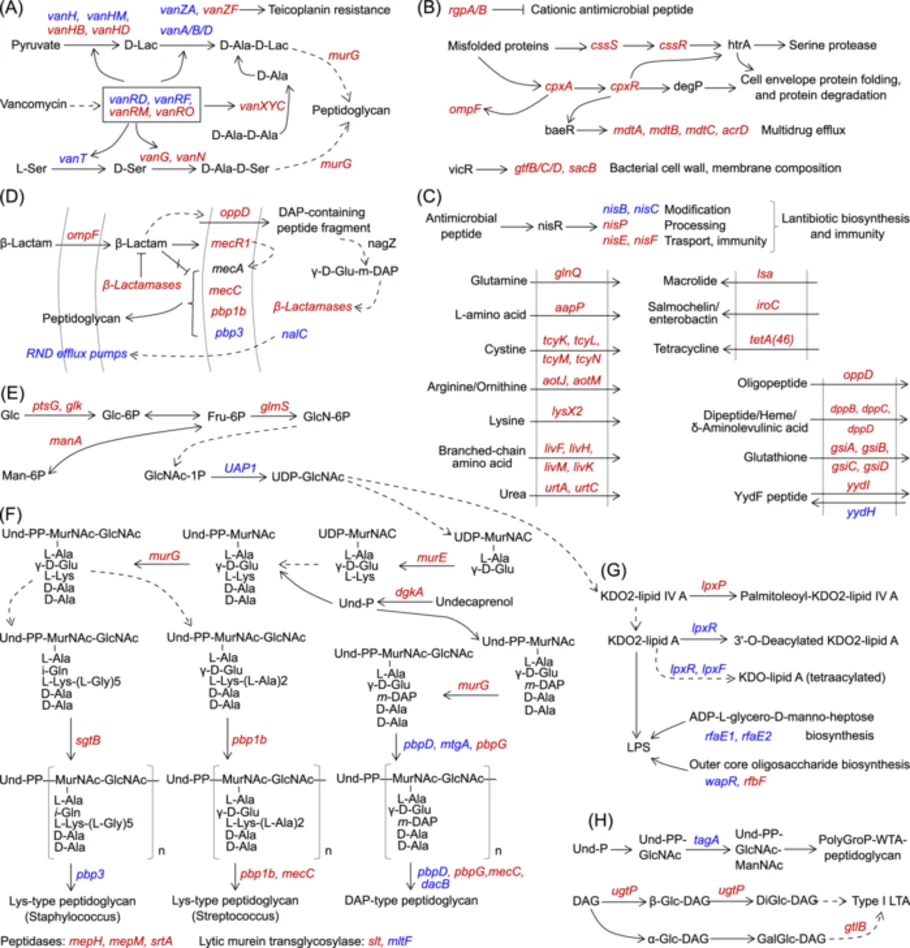
Molecular pathways involved in antibiotic resistance in type 1 diabetes (T1D) rats. (A) Changes in the pathway of vancomycin resistance. (B) Activation of pathways involved in cationic antimicrobial peptide resistance, CpxR signaling, and cell envelop metabolism. (C) Activation of pathways involved in lantibiotic and other antibiotic metabolism, peptide metabolism, and amino acid import. (D) Activation of beta‐lactam resistance pathway. (E) Changes in amino sugar and nucleotide sugar metabolism. (F) Activation of peptidoglycan metabolism. (G) Changes in lipopolysaccharide (LPS) biosynthesis. (H) Changes in teichoic acid biosynthesis. Blue/red fonts, significantly decreased/increased in T1D rats. LTA, lipoteichoic acid.

Changes in the pathway of vancomycin resistance in gut microbiota of T1D rats were discovered (Figures 2A and S10). Decreases in *vanRD* and *vanRF*, and increases in *vanRM* and *vanRO* indicated disturbances in VanR signaling in T1D rats. Decreases in *vanH*, *vanHM*, *vanA/B/D*, and *vanT*, and increases in *vanHB*, *vanHD*, *vanG*, and *vanN* in T1D rats suggested disturbed synthesis of lipid II with terminal d‐Ala‐d‐Lac and/or d‐Ala‐d‐Ser, which mediated the affinity of lipid II to vancomycin and resultant antibiotic resistance [13]. Besides, an increase in *vanXYC* in T1D rats suggested increases in hydrolysis of the terminal d‐Ala from d‐Ala‐d‐Ala and/or UDP‐MurNAc‐pentapeptide, thus reducing the affinity of lipid II to vancomycin and resultant increases in antibiotic resistance [14].

We found activation of pathways involved in antimicrobial peptide resistance, CpxA/CpxR signaling and its downstream multidrug drug efflux and cell envelop metabolism, lantibiotics biosynthesis and immunity, and transport of antibiotics, peptides, and amino acids in T1D rats (Figure 2B,C and Figures S11–S13). Activation of CpxR signaling could stimulate the expression of *ompF* involved in beta‐lactam import, which induced the production of peptidoglycan precursor peptides and resultant activation of beta‐lactamases and beta‐lactam resistance in T1D rats. Besides, activation of CpxR signaling could promote antibiotic (such as beta‐lactams and antimicrobial peptides) resistance by mediating multidrug efflux pump genes (such as *mdtA/B/C/D*) and peptidoglycan metabolic enzymes (such as transpeptidases and transglycosylases), and in turn, degradation products of peptidoglycans and changes in peptidoglycan structure could affect CpxR signaling and its mediated antibiotic resistance [15, 16]. Moreover, increases in *nisP*, *nisE*, and *nisF* in this study indicated the promoted generation of active lantibiotics, and increases in lantibiotic exports and the immunity in T1D rats [17]. Activation of lantibiotic biosynthesis and immunity suggested the involvement of lantibiotic metabolism in selective expansion of gut antibiotic resistome and species contributions of ARG hosts (mainly belonging to order *Lactobacillales*, class *Bacilli*, phylum *Firmicutes*) in T1D rats [17].

Activation of the beta‐lactam resistance pathway was observed in T1D rats (Figure 2D and Figure S14). Increases in *ompF*, *oppD*, and beta‐lactamase genes suggested the activation of antibiotic import for the hydrolysis by lactamases in T1D rats [18]. Besides, gut microbial *mecR1*, *mecC*, and *pbp1b* were increased, while *pbp3* was decreased in T1D rats, which indicated potential increases in penicillin‐binding proteins with low affinity to beta‐lactams, thus conferring peptidoglycan biosynthesis even in the presence of beta‐lactams. Additionally, penicillin‐binding protein 1 B could form beta‐lactam resistance complex with penicillin‐binding protein 5 and transpeptidase YcbB upon alarmone production, thereby circumventing the action of beta‐lactams and restoring crosslinking function [19]. Moreover, *mecC*‐encoded penicillin‐binding protein 2A could collaborate with other monofunctional glycotransferases to induce high‐level oxacillin resistance upon the inhibition of penicillin‐binding protein 2, which suggested potential collaboration between *mecC*‐encoded penicillin‐binding protein 2A and other monofunctional glycotransferases to confer resistance to beta‐lactams and other antibiotics in the case of penicillin‐binding protein 2 inactivation [20].

Changes in pathways related to cell envelop metabolism in T1D rats were further investigated (Figure 2E–H and Figures S15–S18). Most gut microbial genes involved in lipid II biosynthesis, such as *glmS*, *dgkA*, and *murG*, were increased in T1D rats, indicating increases in lipid II biosynthesis (Figure 2E,F). Additionally, increases in *pbp1b* and *mecC* suggested increases in lys‐type peptidoglycans (*Streptococcus*) biosynthesis in T1D rats (Figure 2F). Besides, disturbed gut microbial biosynthesis of lys‐type peptidoglycans (*Staphylococcus*) and DAP‐type peptidoglycans in T1D rats could be evinced by increases in *sgtB*, *pbpG*, and *mecC*, and decreases in *pbp3*, *pbpD*, *mtgA*, and *dacB* (Figure 2F). Additionally, increases in genes encoding peptidases (such as *mepH*, *mepM*, and *srtA*) indicate increases in peptidoglycan modifications by peptidases in T1D rats (Figure 2F). Moreover, Changes in lipopolysaccharide and teichoic acid biosynthesis were also observed in T1D rats (Figure 2G,H).

## 5 CONCLUSION

In conclusion, activation of ARGs involved in antimicrobial peptide resistance, beta‐lactam resistance, vancomycin resistance, CpxR signaling, lantibiotic biosynthesis, and immunity, and transports of peptides and amino acids promotes the synthesis of peptidoglycans and their products, and antimicrobial peptides, which leads to intestinal inflammation, even systemic inflammation, and microbial immunity, thereby promoting T1D (Graphical abstract). Besides, host inflammation and microbial immunity facilitate selective expansion of gut microbes and antibiotic resistome. This study provides novel perspectives and potential intervention targets for studies on the emergence and dissemination of gut microbial ARGs in T1D conditions, roles of gut antibiotic resistome in T1D, and personalized antibiotic administration in T1D patients. As potential intervention targets, key ARG hosts and underlying pathways discovered in this study and their dynamics will be considered in subsequent studies.

## AUTHOR CONTRIBUTIONS

**Guozhu Ye**: Conceptualization; formal analysis; data curation; investigation; funding acquisition; visualization; writing—original draft; writing—review and editing. **Yifang Duan**: Conceptualization; formal analysis; data curation; investigation; visualization. **Haining Huang**: Investigation. **Guoyou Chen**: Investigation. **Minghui Li**: Investigation. **Ricardo David Avellán‐Llaguno**: Investigation. **Qiansheng Huang**: Supervision; funding acquisition.

## CONFLICT OF INTEREST STATEMENT

The authors declare no conflicts of interest.

## ETHICS STATEMENT

All animal procedures were approved by the Animal Ethics Committee of Harbin Medical University‐Daqing (approval number, HMUDQ20240110011). Informed consents from human participants were obtained in the study [12].

## Supporting information


**Figure S1.** Pathophysiological alterations in type 1 diabetes (T1D) rats.
**Figure S2.** Changes in gut antibiotic resistome and relevant resistance mechanisms in type 1 diabetes (T1D) rats.
**Figure S3.** Changes in other antibiotic resistance gene (ARG) subtypes in type 1 diabetes (T1D) rats.
**Figure S4.** Strong correlations among changes in gut antibiotic resistome in type 1 diabetic rats.
**Figure S5.** Selective expansion of gut antibiotic resistome and its health risks in type 2 diabetes (T2D) patients.
**Figure S6.** Correlations of gut antibiotic resistome with the microbial community from the phylum to species.
**Figure S7.** Species contributions of gut microbiota at the phylum level to antibiotic resistance gene (ARG) types in type 1 diabetes (T1D) rats.
**Figure S8.** Heat map plot of species contributions of gut microbiota at the species level to antibiotic resistance gene (ARG) types in type 1 diabetic rats.
**Figure S9.** Changes in species contributions of gut microbiota at the species level to antibiotic resistance gene types in type 1 diabetes (T1D) rats.
**Figure S10.** Changes in the pathway of vancomycin resistance in type 1 diabetic rats.
**Figure S11.** Changes in the pathway of cationic antimicrobial peptide resistance in type 1 diabetic rats.
**Figure S12.** Changes in the pathway of two‐component system in type 1 diabetic rats.
**Figure S13.** Changes in the pathway of ABC transporters in type 1 diabetic rats.
**Figure S14.** Changes in the pathway of beta‐lactam resistance in type 1 diabetic rats.
**Figure S15.** Changes in the pathway of amino sugar and nucleotide sugar metabolism in type 1 diabetic rats.
**Figure S16.** Changes in the pathway of peptidoglycan biosynthesis in type 1 diabetic rats.
**Figure S17.** Changes in the pathway of lipopolysaccharide biosynthesis in type 1 diabetic rats.
**Figure S18.** Changes in the pathway of teichoic acid biosynthesis in type 1 diabetic rats.

**Table S1.** Changes in serum biochemical indexes in type 1 diabetic rats.
**Table S2.** Differential antibiotic resistance genes in type 1 diabetic rats.
**Table S3.** Differential antibiotic resistance genes in type 2 diabetic patients.
**Table S4.** Differential genes in type 1 diabetic rats.

## Data Availability

The data that support the findings of this study are available from the corresponding author upon reasonable request. Raw sequencing data on T1D rats in Fast.gz format have been uploaded to Science Data Bank (https://www.scidb.cn/en/s/QVJFva). Besides, raw sequencing data on T2D patients were downloaded from the NCBI SRA database under the accession number SRA045646 (https://www.ncbi.nlm.nih.gov/sra/?term=SRA045646). Supplementary materials (methods, figures, tables, graphical abstract, slides, videos, Chinese translated version, and update materials) may be found in the online DOI or iMeta Science http://www.imeta.science/imetaomics/.
