# Supplementary material for: Selective expansion of gut antibiotic resistome and underlying pathways involved in type 1 diabetes
Source: IMetaOmics. 2025 Mar 6;2(2):e70007. doi: 10.1002/imo2.70007 (PMC12806347; doi:10.1002/imo2.70007)
Supplement: Supplementary file 1 — Figure S1. Pathophysiological alterations in type 1 diabetes (T1D) rats. Figure S2. Changes in gut antibiotic resistome and relevant resistance mechanisms in type 1 diabetes (T1D) rats. Figure S3. Changes in other antibiotic resistance gene (ARG) subtypes in type 1 diabetes (T1D) rats. Figure S4. Strong correlations among changes in gut antibiotic resistome in type 1 diabetic rats. Figure S5. Selective expansion of gut antibiotic resistome and its health risks in type 2 diabetes (T2D) patients. Figure S6. Correlations of gut antibiotic resistome with the microbial community from the phylum to species. Figure S7. Species contributions of gut microbiota at the phylum level to antibiotic resistance gene (ARG) types in type 1 diabetes (T1D) rats. Figure S8. Heat map plot of species contributions of gut microbiota at the species level to antibiotic resistance gene (ARG) types in type 1 diabetic rats. Figure S9. Changes in species contributions of gut microbiota at the species level to antibiotic resistance gene types in type 1 diabetes (T1D) rats. Figure S10. Changes in the pathway of vancomycin resistance in type 1 diabetic rats. Figure S11. Changes in the pathway of cationic antimicrobial peptide resistance in type 1 diabetic rats. Figure S12. Changes in the pathway of two‐component system in type 1 diabetic rats. Figure S13. Changes in the pathway of ABC transporters in type 1 diabetic rats. Figure S14. Changes in the pathway of beta‐lactam resistance in type 1 diabetic rats. Figure S15. Changes in the pathway of amino sugar and nucleotide sugar metabolism in type 1 diabetic rats. Figure S16. Changes in the pathway of peptidoglycan biosynthesis in type 1 diabetic rats. Figure S17. Changes in the pathway of lipopolysaccharide biosynthesis in type 1 diabetic rats. Figure S18. Changes in the pathway of teichoic acid biosynthesis in type 1 diabetic rats. [file IMO2-2-e70007-s002.docx]

**Supporting information to Selective expansion of gut antibiotic resistome and underlying pathways involved in type 1 diabetes**

**Running title:** Distinctive gut antibiotic resistome in type 1 diabetes

Guozhu Ye^1,*,#^, Yifang Duan^1,#^, Haining Huang^1^, Guoyou Chen^2^, Minghui Li^2^, Ricardo David Avellán-Llaguno^2^, Qiansheng Huang^1^

^1^Xiamen Key Laboratory of Indoor Air and Health, Center for Excellence in Regional Atmospheric Environment, Key Lab of Urban Environment and Health, Institute of Urban Environment, Chinese Academy of Sciences, Xiamen 361021, China.

^2^College of pharmacy, Daqing Campus, Harbin Medical University, Daqing, 163319, China.

^#^ These authors contributed equally: Guozhu Ye, Yifang Duan.

*Correspondence: [gzye@iue.ac.cn](mailto:gzye@iue.ac.cn) (Guozhu Ye), Institute of Urban Environment, Chinese Academy of Sciences, Xiamen 361021, China

**MATERIALS AND METHODS**

**Materials**

Streptozotocin ( ≥ 98%) was purchased from Innochem (Beijing) Technology Co., Ltd (Beijing, China). Male Sprague Dawley rats (6 weeks old) were obtained from Changchun Yisi Laboratory Animal Technology Co., Ltd. (Changchun, China). Assay kits for glucose, total cholesterol, low-density lipoprotein cholesterol, high-density lipoprotein cholesterol, triglyceride, urate, urea, gamma-glutamyl transferase, aspartate transaminase, and alanine transaminase were products from Shenzhen Mindray Animal Medical Technology Co., Ltd. (China).

**Animal experiments and sample collection**

Animal experiments in this study were carried out with the approval of the Animal Ethics Committee of Harbin Medical University-Daqing (approval number, HMUDQ20240110011), and in compliance with the ARRIVE (Animal Research: Reporting of In Vivo Experiments) guidelines and the Guidelines for the Care and Use of Laboratory Animals [[1](#_ENREF_1)]. Rats were housed in a specific pathogen-free environment with a 12-h light/12-h dark cycle, a relative humidity of 60 ± 10%, and a temperature of 20 ± 2 ^0^C. Following one-week adaptive feeding, rats were randomly divided into the control and diabetes group, with 8 male rats in each group. Sprague-Dawley rats were intraperitoneally injected with 60 mg/kg of streptozotocin once to destroy pancreatic islet beta cells via inducing DNA and protein alkylation, and then triggered insulin-dependent type 1 diabetes (T1D) [[2](#_ENREF_2)]. Rats in the control group were treated with an equal volume of deionized water at one time. On day 8, the rat blood was taken from the tail vein after 12-h fasting, and then the fasting blood glucose was detected by a blood glucose meter (Performa, ROCHE, Switzerland). As expected, blood glucose contents of rats in the diabetes group were more than 20 mmol/L, which indicated that the disease model was successfully established. On day 15, after another week of stabilization and progression of the disease, rats from each group were anesthetized with sodium pentobarbital and then sacrificed. Serum and stool samples were collected and stored immediately at -80 °C for subsequent determination of biochemical indexes and metagenomic analysis (*n* = 4 per group), respectively.

All experiments were conducted by the trained personnel to ensure compliance with animal welfare regulations and to minimize the number of animals used, in line with the principles of replacement, reduction, and refinement. Efforts were made to minimize animal distress throughout the study. Rats were also monitored for signs of distress, and any rat showing abnormal behavior or signs of severe discomfort was immediately removed from the study and given appropriate care.

**Hematoxylin and eosin staining of pancreatic tissues**

The pancreatic and colonic tissues were collected from rats in each group. After fixation in 4% paraformaldehyde solution, the tissue was cut into 4-μm sections, and then embedded with paraffin. Subsequently, each sample was cut into multiple sections prior to the staining with hematoxylin and eosin for histological analysis.

**Metagenomic analysis of T1D rats**

The Fast DNA Stool Mini Kit (Qiagen, Germany) was used to exact microbial DNAs from each stool sample of approximately 0.3 g. DNA sequencing and the library construction were carried out via Illumina platform at Majorbio Co., Ltd. (Shanghai, China). The fastp tool (https://github.com/OpenGene/fastp, version 0.20.1) was employed to remove adapter sequences from both the 3' and 5' ends of the reads. Subsequently, reads with an average quality score below 20 were filtered out, and those shorter than 50 bp following quality trimming were excluded. Following the data filtering and removal of the host DNA sequences, the high-quality reads were retained. Assembly of clean reads was performed by the assembler MEGAHIT (version 1.1.2) via a multiple k-mer size strategy based on succinct de Bruijn graphs [[3](#_ENREF_3)]. Contigs of Prodigal (version 2.6.3) was applied for predicting open reading frames (ORFs) [[4](#_ENREF_4)]. Contigs greater than or equal to 300 bp were retained. The ORF with a length of 100 bp or more was screened, and then translated into the amino acid sequence. A non-redundant gene catalogue combining all of the predicted genes was generated by CD-HIT software (version 4.6.1) [[5](#_ENREF_5)]. ORFs carrying annotated antibiotic resistance genes (ARGs) were defined as ARG-like ORFs. Antibiotic resistance contigs were defined as contigs that carrying at least one ARG-like ORF. Based on the NCBI-NR database (version 202209), taxonomic annotation was performed on ORFs in antibiotic resistance contigs using BLASTP implemented in DIAMOND (version 2.0.13), with the parameter set to an e-value of ≤ 1E^-5^ [[6](#_ENREF_6)]. Gene abundances were calculated using the values of RPKM (reads per kilobase million) [[7](#_ENREF_7)]. ARGs were identified by blasting protein sequences against the Comprehensive Antibiotic Research Database (version 3.0.9) using stringent cut offs (identity > 80%, and alignment length > 25). To map the pathway, the non-redundant gene catalogue was compared against Kyoto Encyclopedia of Genes and Genomes databases (BLASTP, e-value ≤ 1E^-5^) using DIAMOND software. Subsequently, KEGG Orthology Based Annotation System 2.0 was used for the functional annotation [[8](#_ENREF_8)].

**Metagenomic analysis of diabetic patients**

Metagenomic sequencing data of 145 individuals, including 74 normal controls and 71 type 2 diabetic (T2D) patients, were downloaded from the NCBI SRA database (accession number, SRA045646) [[9](#_ENREF_9)]. Raw FastQ files were extracted using the SRA Toolkit (version 2.11.0). Quality control was performed on the raw data using fastp (version 0.20.1) to remove adapter sequences and low-quality reads. The quality filtering parameters were set to a minimum quality threshold of Q20 and a minimum sequence length of 50 bp, while other parameters were kept as defaults. High-quality sequences obtained after quality control were de novo assembled using MEGAHIT (version 1.2.9). The assembly parameters were set with a k-mer range from 35 to 95, a step size of 20, and a minimum contig length of 500 bp to ensure the generation of longer contigs for downstream analysis. Genes were predicted from the assembled contigs using Prodigal (version 2.6.3) in metagenomic mode. The predicted gene sequences were clustered using CD-HIT (version 4.8.1) with a sequence similarity threshold of 95% (identity threshold = 0.95) and an alignment coverage threshold of 90% (alignment coverage = 0.9) to construct a non-redundant gene catalog. The non-redundant gene catalog was indexed using Bowtie2 (version 2.4.2), and quality-controlled reads were mapped to the catalog. The resulting SAM files were converted to BAM format, sorted, and quantified using SAMtools (version 1.11). Gene abundance was calculated using the values of RPKM [[7](#_ENREF_7)]. Functional annotation of the non-redundant gene catalog was performed to identify antibiotic resistance genes using the RGI tool (version 6.0.1) based on the Comprehensive Antibiotic Resistance Database. The DIAMOND mode was used for the alignment, with a similarity threshold of 95%, and the annotated antibiotic resistance genes were output as the final results.

**Statistical analysis**

Principal component analysis and spearman correlation analysis was conducted by MetaboAnalyst 6.0 [[10](#_ENREF_10)]. MannWhitney U test (two-tailed) and heat map plot were carried out via MeV 4.9.0 [[11](#_ENREF_11)]. Correlation network analysis were carried out using Cytoscape 2.8.2 [[12](#_ENREF_12)]. Principal coordinates analysis, analysis of species contributions to ARG types/subtypes, analysis of main functions possessed by the species were carried out by the Majorbio cloud platform. The statistical significance level was lower than 0.05. Pathway mapping of differential genes (Table S4, *p* < 0.05, two-tailed Man-Whitney U test) was executed via Kyoto Encyclopedia of Genes and Genomes.

Figure S1 Pathophysiological alterations in type 1 diabetes (T1D) rats. (A) Hematoxylin and eosin staining of morphological changes in pancreatic tissues. (B) Changes in serum biochemical indexes. HDL-C, high-density lipoprotein cholesterol; LDL-C, low-density lipoprotein cholesterol; TC, total cholesterol; ALT, alanine transaminase; AST, aspartate transaminase; GGT, gamma-glutamyl transferase. *, *p* < 0.05, two-tailed Man-Whitney U test. C, the control group. (C) Hematoxylin and eosin staining of morphological changes in colonic tissues.

Figure S2 Changes in gut antibiotic resistome and relevant resistance mechanisms in type 1 diabetes (T1D) rats. (A) Changes in abundances of gut microbial antibiotic resistance gene (ARG) types. (B) Changes in antibiotic resistance mechanisms of gut microbial ARGs. (C) Changes in gut microbial ARG subtypes. All differential ARGs in gut microbiota were listed (*p* < 0.05, two-tailed Man-Whitney U test). *, *p* < 0.05, two-tailed Man-Whitney U test.

Figure S3 Changes in other antibiotic resistance gene (ARG) subtypes in type 1 diabetes (T1D) rats. (A) Multidrug ARGs. RND antibiotic efflux pump, resistance-nodulation-cell division antibiotic efflux pump. MFS antibiotic efflux pump, major facilitator superfamily antibiotic efflux pump. (B) Aminoglycoside ARGs. (C) Phenicol ARGs. *, *p* < 0.05, two-tailed Man-Whitney U test.

Figure S4 Strong correlations among changes in gut antibiotic resistome in type 1 diabetic rats. The Spearman correlation coefficient was lower or greater than 0.9. Blue/red circles, significantly decreased/increased in diabetic rats; blue/red lines, negation/positive correlations.

Figure S5 Selective expansion of gut antibiotic resistome and its health risks in type 2 diabetes (T2D) patients. (A) Changes in profiles of gut antibiotic resistome. (B) Changes in compositions of gut microbial antibiotic resistance gene (ARG) types. (C) Changes in gut microbial ARG subtypes and their correlations with clinical factors, and T2D risks. All differential ARGs in gut microbiota were listed (*p* < 0.05, two-tailed Man-Whitney U test) in the left heat map. After the unit variance scaling, the mean of each group was used to plot the heat map. *, *p* < 0.05, spearman correlation. Red/blue triangle: significantly elevated/decreased clinical factors in T2D patients. *n* = 74 and 71 in the control and T2D group, respectively. FBG, fasting blood glucose. HBALC, glycosylated hemoglobin HbAlc. TG, triglyceride. HDL, high density lipoprotein. SBP, systolic blood pressure. DBP, diastolic blood pressure. TCHO, total cholesterol. LDL, low density lipoprotein. BMI, body mass index. FINS, fasting serum insulin.

Figure S6 Correlations of gut antibiotic resistome with the microbial community from the phylum to species.

Figure S7 Species contributions of gut microbiota at the phylum level to antibiotic resistance gene (ARG) types in type 1 diabetes (T1D) rats. (A) Species contributions of gut microbiota at the phylum level to ARG types. (B) The main ARG functions in which gut microbial species were involved. (C) Contributions of *Firmicutes* to ARG types. *, *p* < 0.05, two-tailed Man-Whitney U test. C, the control group; D, the diabetic group.

Figure S8 Heat map plot of species contributions of gut microbiota at the species level to antibiotic resistance gene (ARG) types in type 1 diabetic rats. Relevant significant species contributions were all listed (*p* < 0.05, two-tailed Man-Whitney U test). C, the control group; D, the diabetic group.

Figure S9 Changes in species contributions of gut microbiota at the species level to antibiotic resistance gene types in type 1 diabetes (T1D) rats. *, *p* < 0.05, two-tailed Man-Whitney U test.


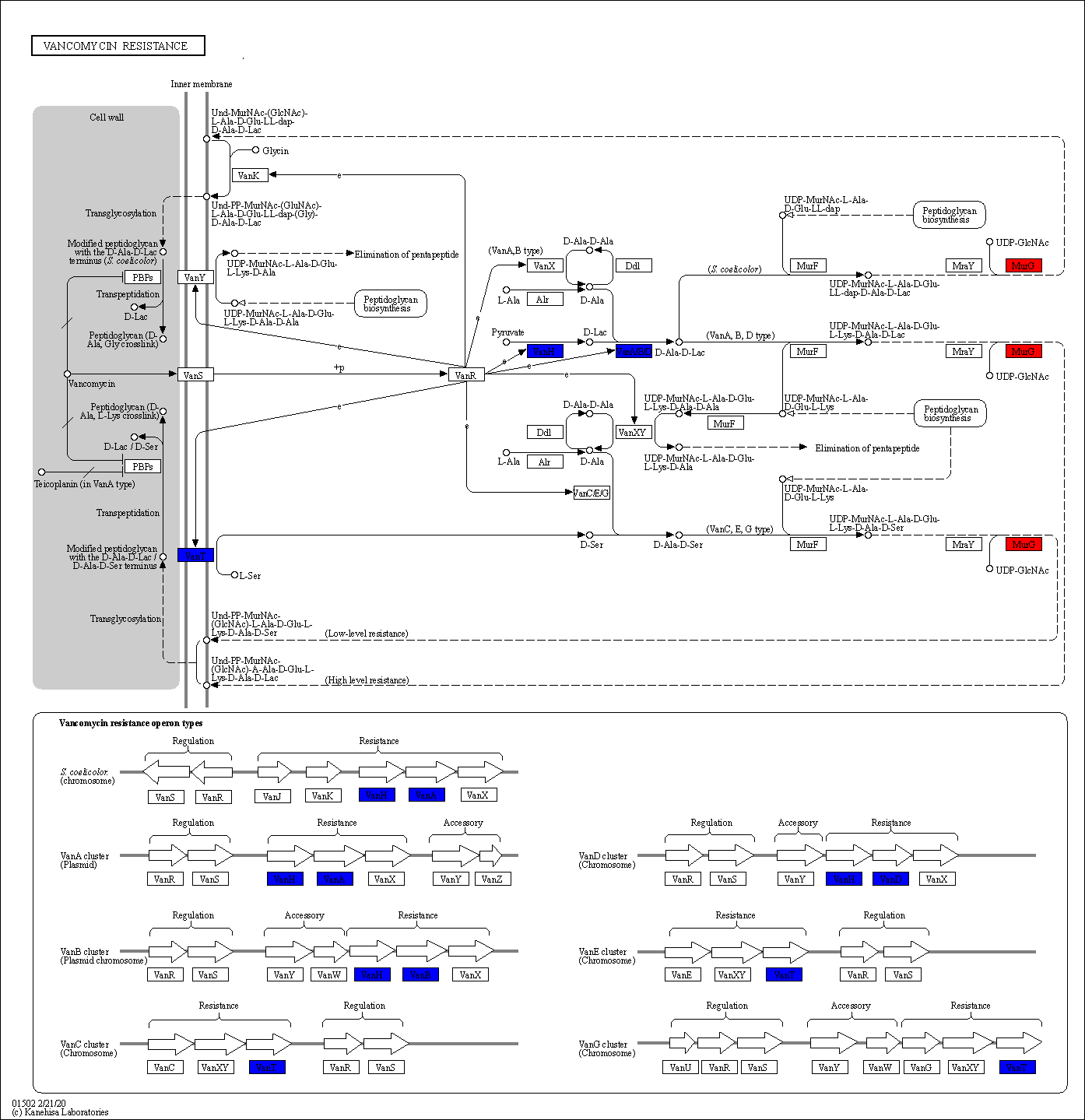


Figure S10 Changes in the pathway of vancomycin resistance in type 1 diabetic rats. Blue/red backgrounds, relevant genes significantly decreased/increased in type 1 diabetic rats (*p* < 0.05, two-tailed Man-Whitney U test).


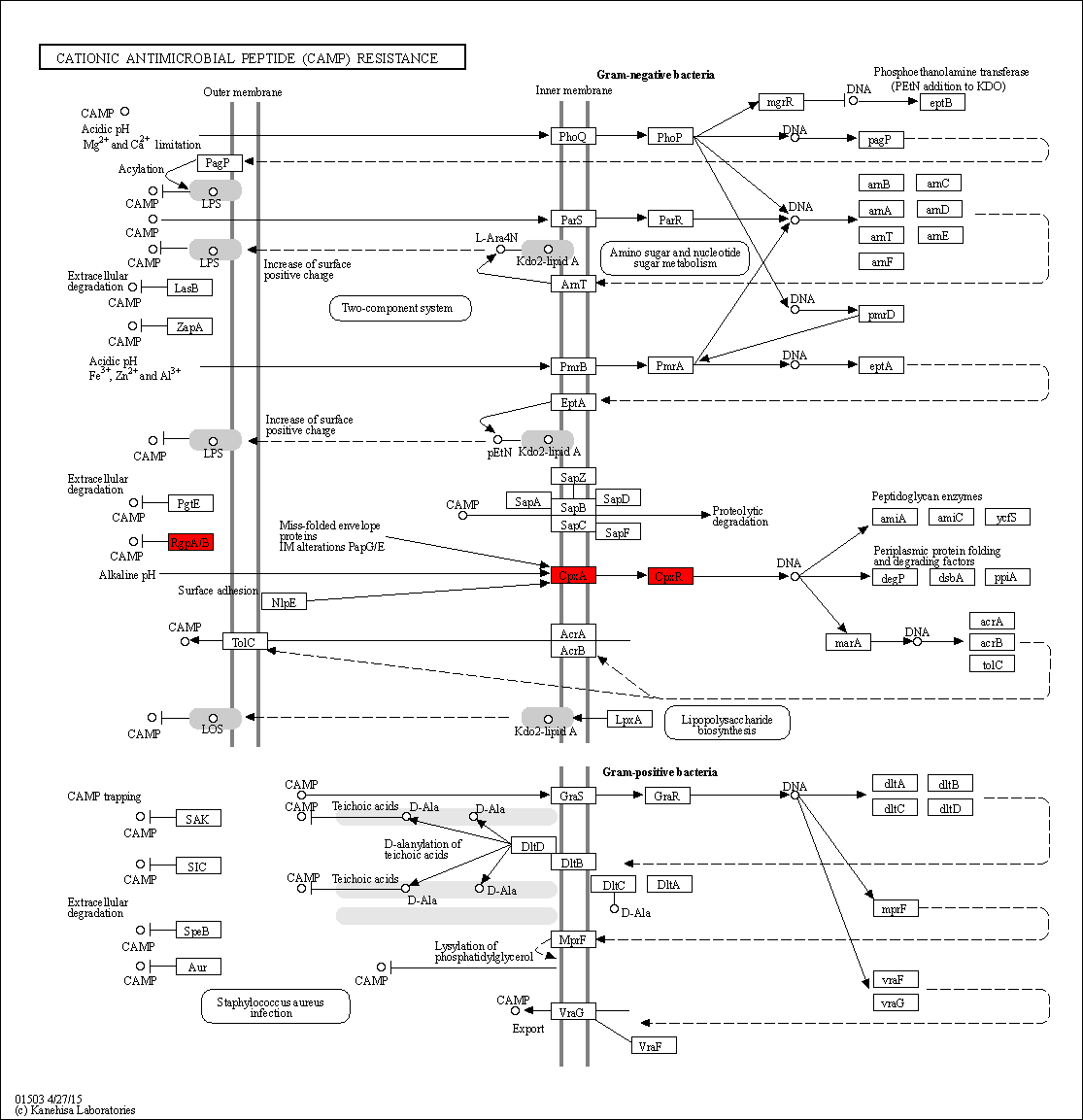


Figure S11 Changes in the pathway of cationic antimicrobial peptide resistance in type 1 diabetic rats. Red backgrounds, relevant genes significantly increased in type 1 diabetic rats (*p* < 0.05, two-tailed Man-Whitney U test).


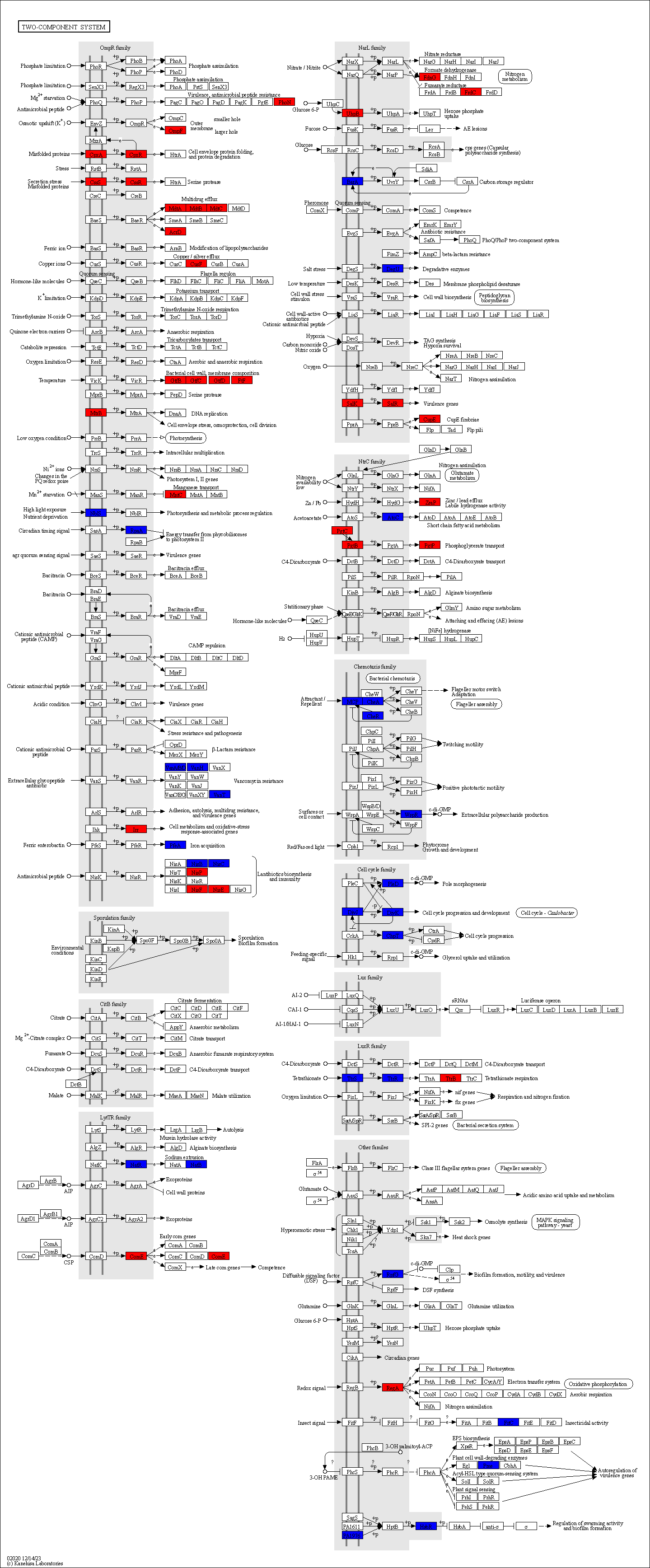


Figure S12 Changes in the pathway of two-component system in type 1 diabetic rats. Blue/red backgrounds, relevant genes significantly decreased/increased in type 1 diabetic rats (*p* < 0.05, two-tailed Man-Whitney U test).


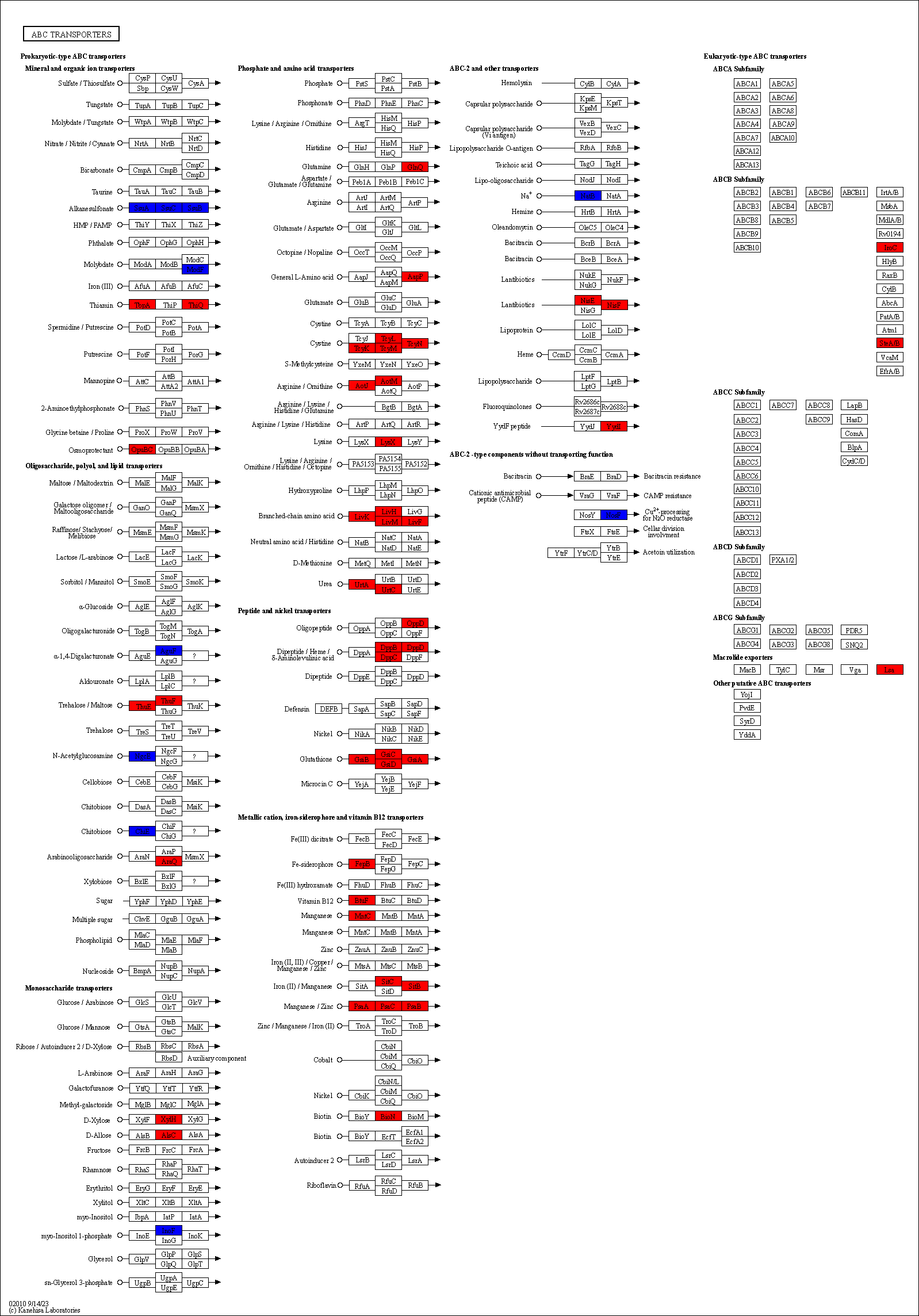


Figure S13 Changes in the pathway of ABC transporters in type 1 diabetic rats. Blue/red backgrounds, relevant genes significantly decreased/increased in type 1 diabetic rats (*p* < 0.05, two-tailed Man-Whitney U test).


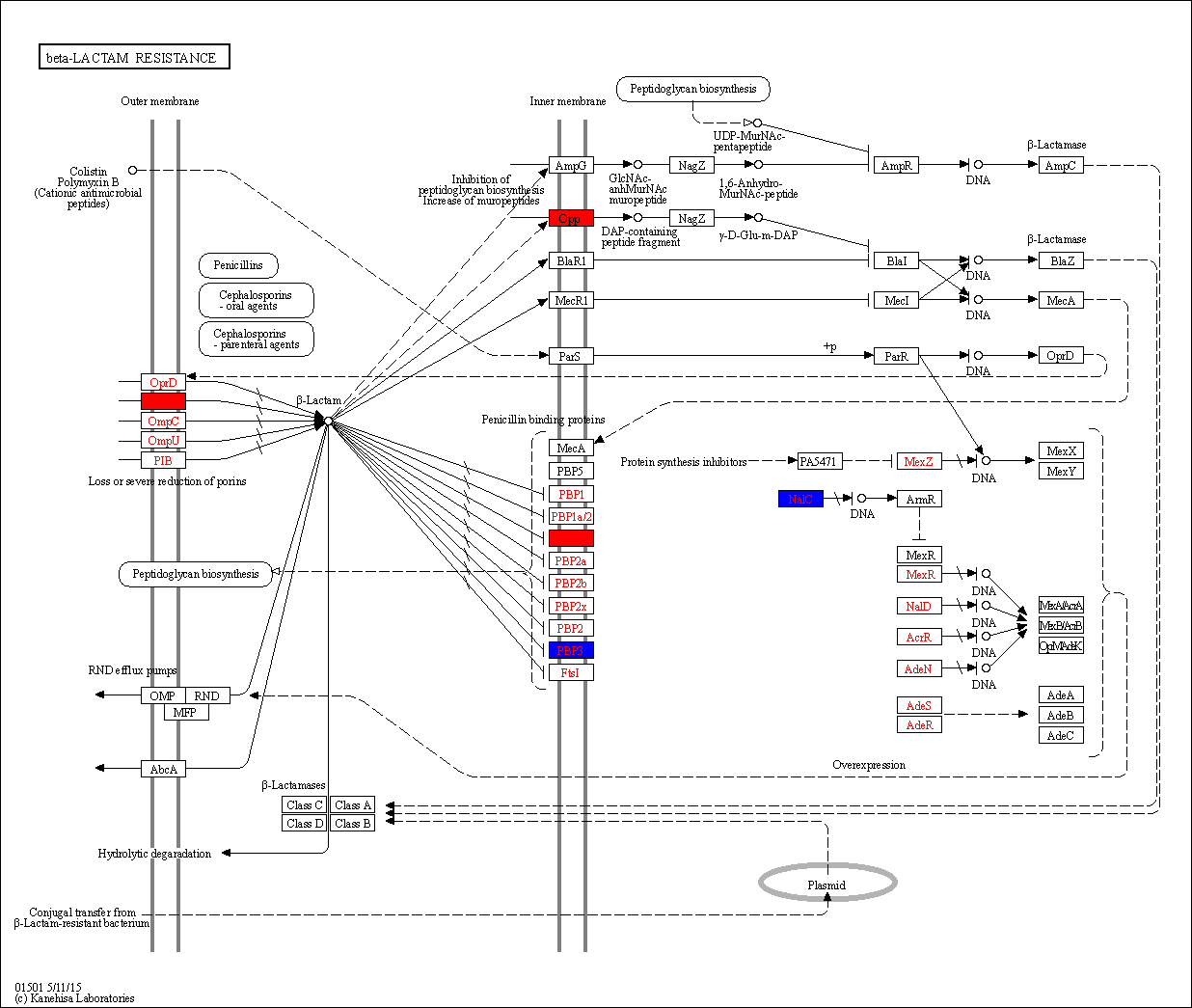


Figure S14 Changes in the pathway of beta-lactam resistance in type 1 diabetic rats. Blue/red backgrounds, relevant genes significantly decreased/increased in type 1 diabetic rats (*p* < 0.05, two-tailed Man-Whitney U test).


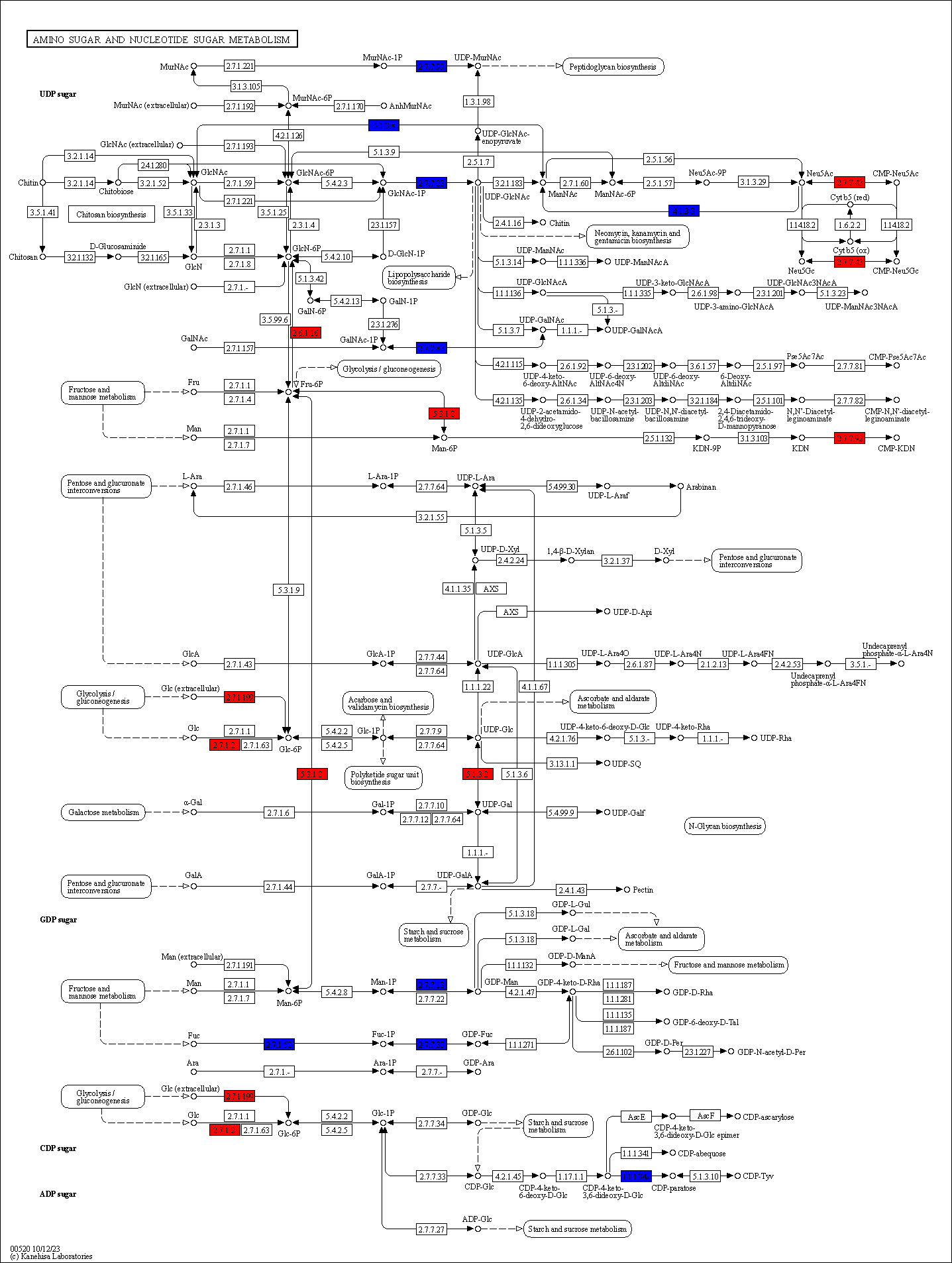


Figure S15 Changes in the pathway of amino sugar and nucleotide sugar metabolism in type 1 diabetic rats. Blue/red backgrounds, relevant genes significantly decreased/increased in type 1 diabetic rats (*p* < 0.05, two-tailed Man-Whitney U test).


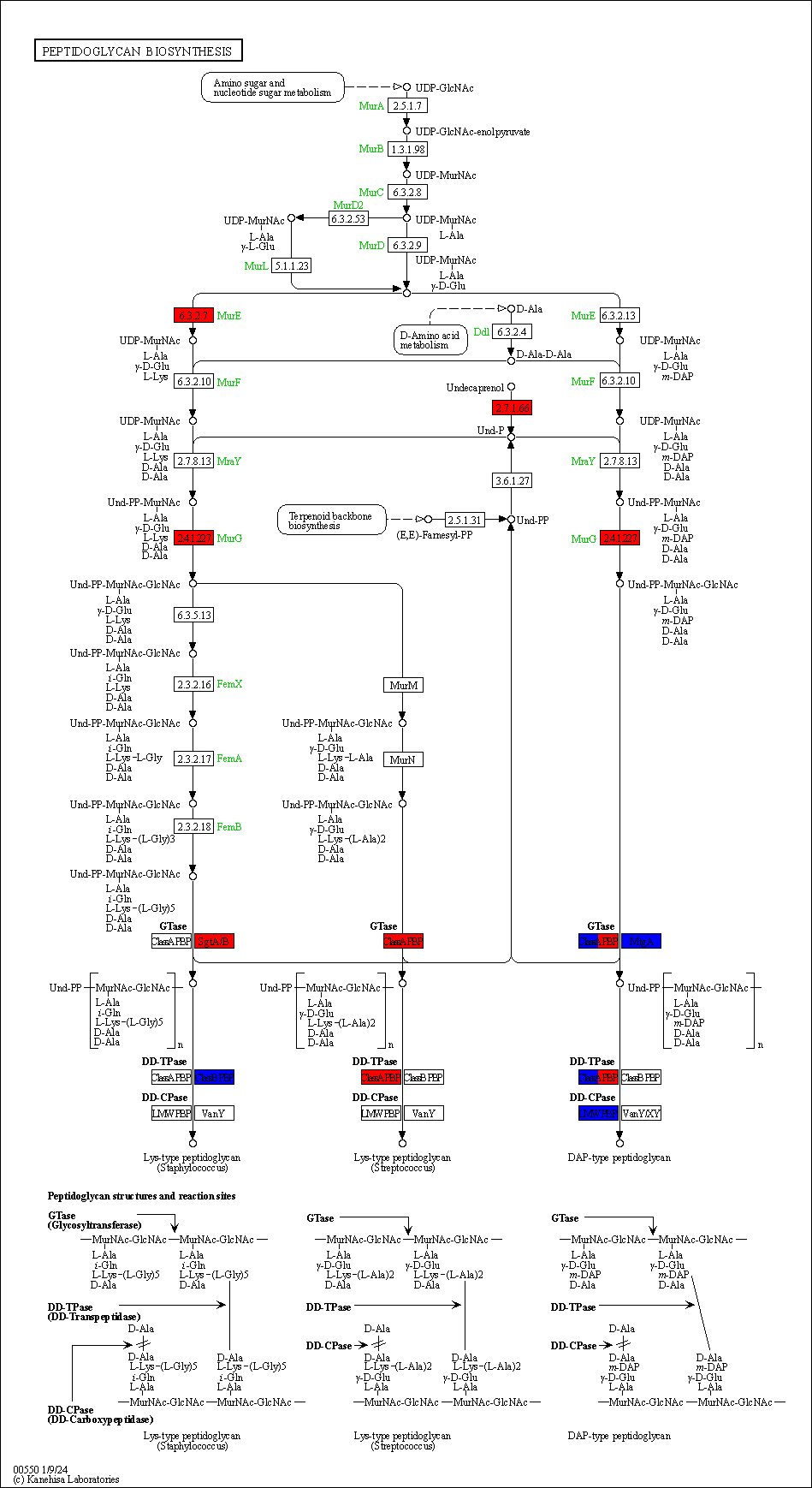


Figure S16 Changes in the pathway of peptidoglycan biosynthesis in type 1 diabetic rats. Blue/red backgrounds, relevant genes significantly decreased/increased in type 1 diabetic rats (*p* < 0.05, two-tailed Man-Whitney U test).


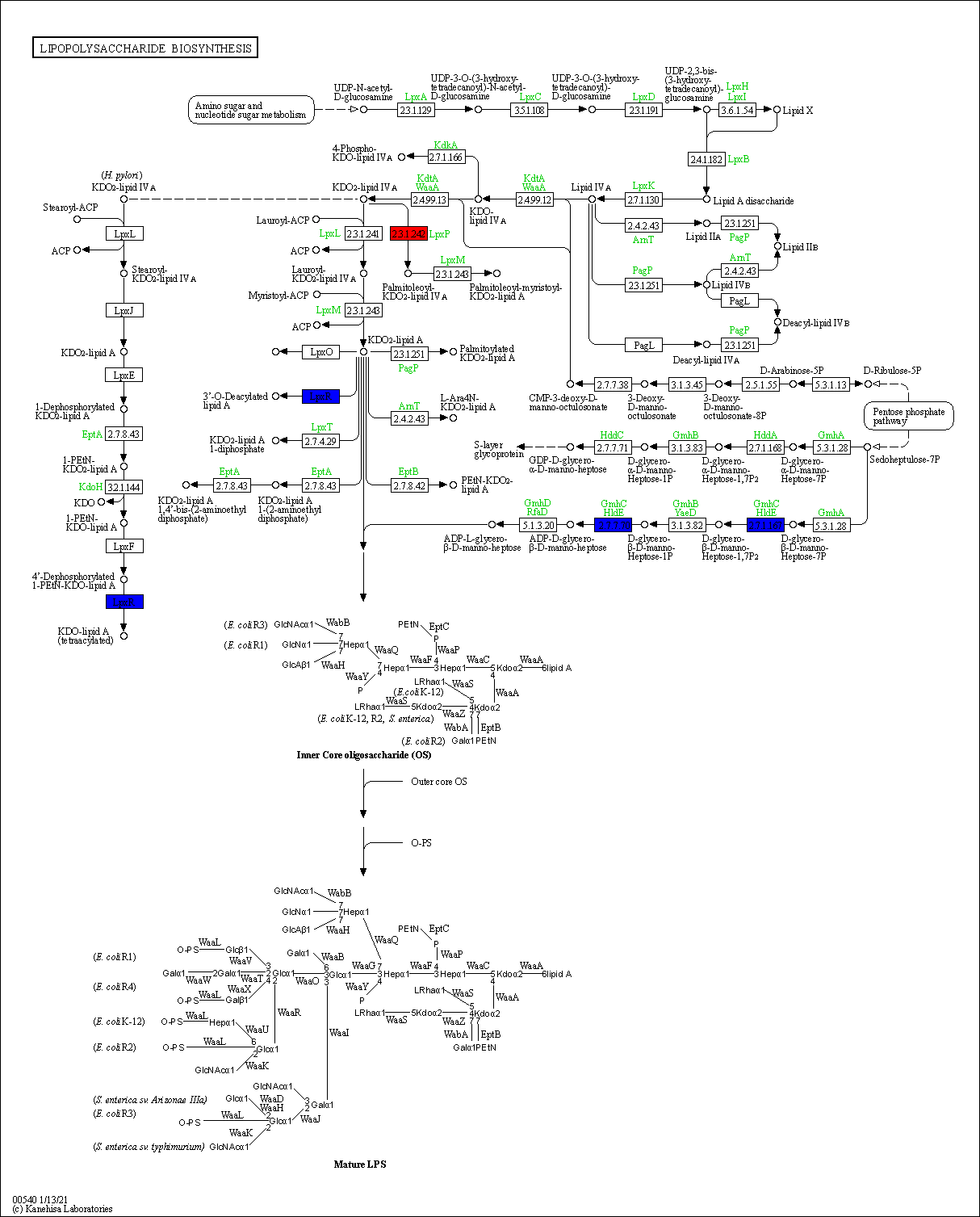


Figure S17 Changes in the pathway of lipopolysaccharide biosynthesis in type 1 diabetic rats. Blue/red backgrounds, relevant genes significantly decreased/increased in type 1 diabetic rats (*p* < 0.05, two-tailed Man-Whitney U test).


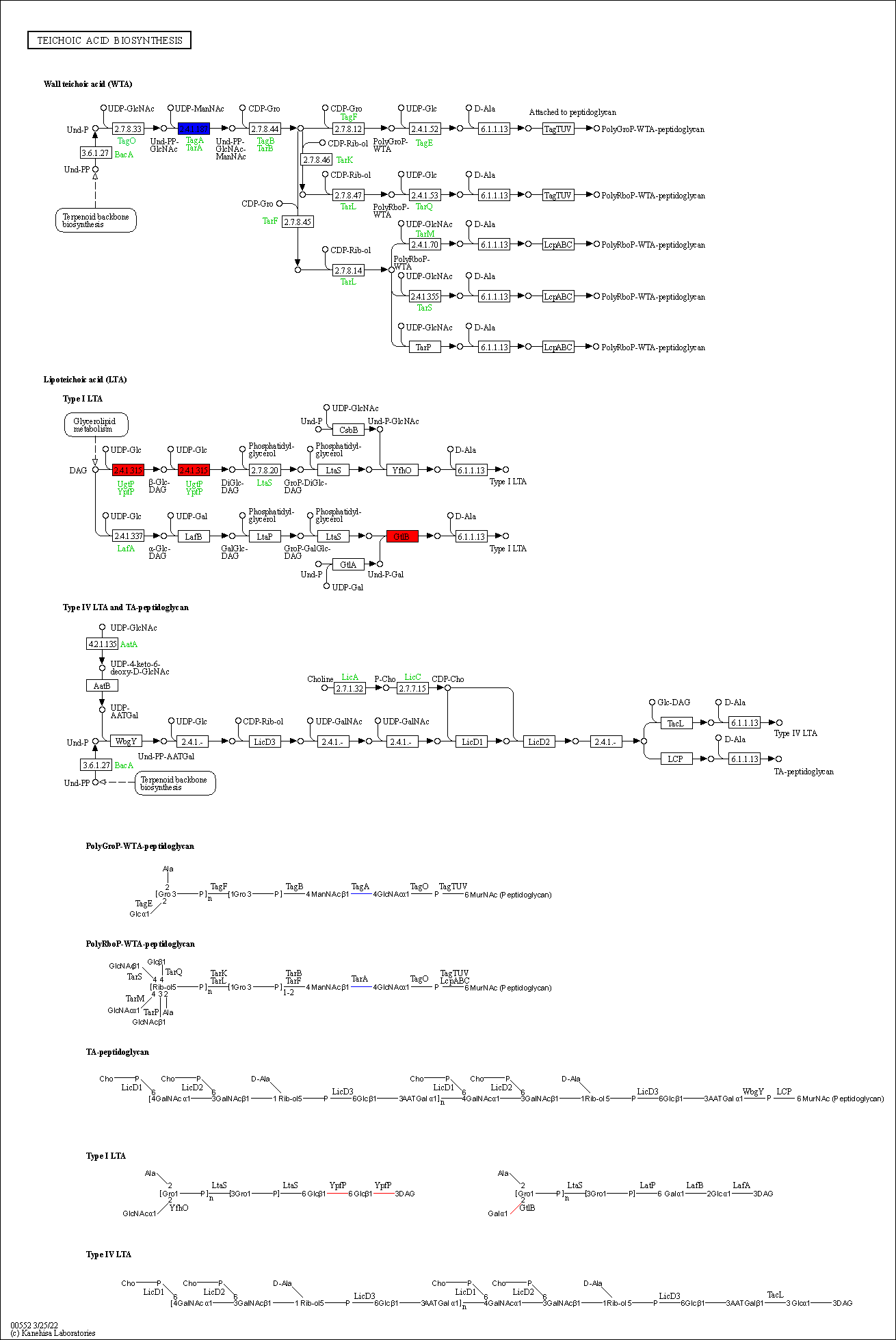


Figure S18 Changes in the pathway of teichoic acid biosynthesis in type 1 diabetic rats. Blue/red backgrounds, relevant genes significantly decreased/increased in type 1 diabetic rats (*p* < 0.05, two-tailed Man-Whitney U test).

**REFERENCES**

1. Kilkenny, Carol, William Browne, Innes C Cuthill, Michael Emerson, Douglas G Altman. 2010. “Animal research: reporting in vivo experiments: the ARRIVE guidelines.” *British Journal Of Pharmacology* 160:1577—1579. <https://doi.org/10.1111/j.1476-5381.2010.00872.x>

2. Lenzen, S. 2008. “The mechanisms of alloxan- and streptozotocin-induced diabetes.” *Diabetologia* 51:216—226. <https://doi.org/10.1007/s00125-007-0886-7>

3. Li, Dinghua, Chi-Man Liu, Ruibang Luo, Kunihiko Sadakane, Tak-Wah Lam. 2015. “MEGAHIT: an ultra-fast single-node solution for large and complex metagenomics assembly via succinct de Bruijn graph.” *Bioinformatics* 31:1674—1676. <https://doi.org/10.1093/bioinformatics/btv033>

4. Hyatt, Doug, Gwo-Liang Chen, Philip F. Locascio, Miriam L. Land, Frank W. Larimer, Loren J. Hauser. 2010. “Prodigal: prokaryotic gene recognition and translation initiation site identification.” *BMC Bioinformatics* 11:119. <https://doi.org/10.1186/1471-2105-11-119>

5. Fu, Limin, Beifang Niu, Zhengwei Zhu, Sitao Wu, Weizhong Li. 2012. “CD-HIT: accelerated for clustering the next-generation sequencing data.” *Bioinformatics* 28:3150—3152. <https://doi.org/10.1093/bioinformatics/bts565>

6. Buchfink, Benjamin, Chao Xie, Daniel H. Huson. 2015. “Fast and sensitive protein alignment using DIAMOND.” *Nature Methods* 12:59—60. <https://doi.org/10.1038/nmeth.3176>

7. Lawson, Christopher E., Sha Wu, Ananda S. Bhattacharjee, Joshua J. Hamilton, Katherine D. McMahon, Ramesh Goel, Daniel R. Noguera. 2017. “Metabolic network analysis reveals microbial community interactions in anammox granules.” *Nature Communications* 8:15416. <https://doi.org/10.1038/ncomms15416>

8. Xie, Chen, Xizeng Mao, Jiaju Huang, Yang Ding, Jianmin Wu, Shan Dong, Lei Kong, Ge Gao, Chuan-Yun Li, Liping Wei. 2011. “KOBAS 2.0: a web server for annotation and identification of enriched pathways and diseases.” *Nucleic Acids Research* 39:W316—W322. <https://doi.org/10.1093/nar/gkr483>

9. Qin, Junjie, Yingrui Li, Zhiming Cai, Shenghui Li, Jianfeng Zhu, Fan Zhang, Suisha Liang, et al.. 2012. “A metagenome-wide association study of gut microbiota in type 2 diabetes.” *Nature* 490:55—60. <https://doi.org/10.1038/nature11450>

10. Ewald, Jessica D., Guangyan Zhou, Yao Lu, Jelena Kolic, Cara Ellis, James D. Johnson, Patrick E. Macdonald, Jianguo Xia. 2024. “Web-based multi-omics integration using the Analyst software suite.” *Nature Protocols* 19:1467—1497. <https://doi.org/10.1038/s41596-023-00950-4>

11. Saeed, Alexander I., Nirmal K. Bhagabati, John C. Braisted, Wei Liang, Vasily Sharov, Eleanor A. Howe, Jianwei Li, Mathangi Thiagarajan, Joseph A. White, John Quackenbush. 2006. “TM4 microarray software suite.” *Methods In Enzymology* 411:134—193. <https://doi.org/10.1016/S0076-6879(06)11009-5>

12. Cline, Melissa S., Michael Smoot, Ethan Cerami, Allan Kuchinsky, Nerius Landys, Chris Workman, Rowan Christmas, et al. 2007. “Integration of biological networks and gene expression data using Cytoscape.” *Nature Protocols* 2:2366—2382. <https://doi.org/10.1038/nprot.2007.324>
